# Polymorphic mimicry - When Herpes Isn’t Just Herpes: Genital Herpes Mimicking Secondary Syphilis: A Case Report

**DOI:** 10.51894/001c.165152

**Published:** 2026-08-27

**Authors:** Andrew Vierra, Leena Jamal, Khadijah Bokhari, Adam Makki, Taylor Liebert, Abdullah Bokhari

**Affiliations:** 1 Emergency Medicine Residency Program McLaren Oakland Hospital, Pontiac, MI, USA; 2 College of Osteopathic Medicine Michigan State University, East Lansing MI, USA; 3 Emergency Medicine McLaren Health Care; 4 College of Osteopathic Medicine Michigan State University

**Keywords:** Herpes Simplex Virus-2 (HSV-2), Erythema Multiforme, Secondary Syphilis, Genital Ulcers, Osteopathic Medicine

## Abstract

**Background:**

Herpes simplex virus type 2 (HSV-2) is a highly prevalent sexually transmitted infection (STI), affecting approximately 13% of the global population. While classic presentations involve painful vesicular clusters, HSV-2 can present atypically or trigger immune-mediated reactions like erythema multiforme (EM). These polymorphic presentations can mimic other STIs, particularly secondary syphilis, leading to diagnostic momentum and delayed appropriate therapy.

**Case presentation:**

A 47-year-old male with a history of recurrent genital ulcers presented to the emergency department with worsening penile lesions, anal pain, and a new onset rash on his palms and soles. He had been treated for presumed syphilis two months prior. Examination revealed vesiculopustular genital lesions, inguinal lymphadenopathy, perianal ulcers resembling fissures, and targetoid lesions on the extremities. The clinical triad of genital ulcers, anal pathology, and a palmoplantar rash strongly suggested secondary syphilis. However, syphilis serology returned negative. Polymerase chain reaction (PCR) testing confirmed HSV-2. The acral rash was diagnosed as EM secondary to HSV.

**Diagnosis and Outcome:**

The patient was diagnosed with HSV-2 proctitis and HSV-associated erythema multiforme. He was treated with high-dose valacyclovir. At the two-week follow-up, genital and anal lesions had healed, and the systemic rash had resolved.

**Conclusion:**

HSV-associated erythema multiforme can produce a clinical picture indistinguishable from secondary syphilis. Emergency clinicians must maintain a broad differential for genital ulcers with acral rashes. PCR testing is superior to syndromic diagnosis in complex presentations to prevent unnecessary antibiotic exposure and ensure timely antiviral management.

## INTRODUCTION

Herpes simplex virus type 2 (HSV-2) remains one of the most common sexually transmitted infections (STIs) worldwide. In the United States alone, the prevalence of HSV-2 in persons aged 14–49 is estimated at 11.9%. While the “classic” presentation involves painful groupings of vesicles on an erythematous base, the clinical spectrum of HSV is broad. Atypical manifestations can include fissures, hypertrophic plaques, and extragenital lesions, frequently leading to misdiagnosis as chancroid, fungal infections, or trauma.^1–4^

Diagnostic difficulty increases significantly when genital ulcers are accompanied by systemic rashes. Syphilis, often termed “the great imitator,” is the primary consideration for any patient presenting with genital lesions and a rash involving the palms and soles.^5–8^ However, HSV is the most common identified trigger for erythema multiforme (EM), an acute immune-mediated hypersensitivity reaction characterized by targetoid cutaneous lesions.^2,9,10^ When genital HSV acts as the precipitating factor for EM, the resulting syndrome—genital ulcers combined with an acral rash—creates a polymorphic mimicry of secondary syphilis.^5^

We present a case of a 47-year-old male whose recurrent HSV-2 infection, complicated by proctitis and erythema multiforme, was managed as syphilis twice before definitive testing clarified the diagnosis. This report underscores the limitations of syndromic management in atypical STI presentations and discusses the osteopathic implications of treating complex sexually transmitted diseases.

## CASE PRESENTATION

**History** A 47-year-old male presented to the emergency department (ED) reporting a four-day history of painful penile blisters and a spreading rash on his hands and feet. He also endorsed new-onset rectal pain aggravated by bowel movements. The patient reported a medical history significant only for recurrent genital sores over the past six months. He had sought care at multiple urgent cares and EDs for these episodes. Two months prior to the current visit, he was treated with intramuscular benzathine penicillin for presumed syphilis, though he was unsure of the confirmatory test results. He had also received multiple courses of doxycycline without resolution. He reported being sexually active with multiple female partners and used condoms inconsistently. He denied fever, chills, vision changes, or joint pain.

**Physical examination:** On arrival, the patient was afebrile (37.0°C) and hemodynamically stable with a blood pressure of 134/78 mmHg, heart rate of 76 beats per minute, respiratory rate of 16 breaths per minute, and oxygen saturation of 98% on room air.

**Figure 1.**
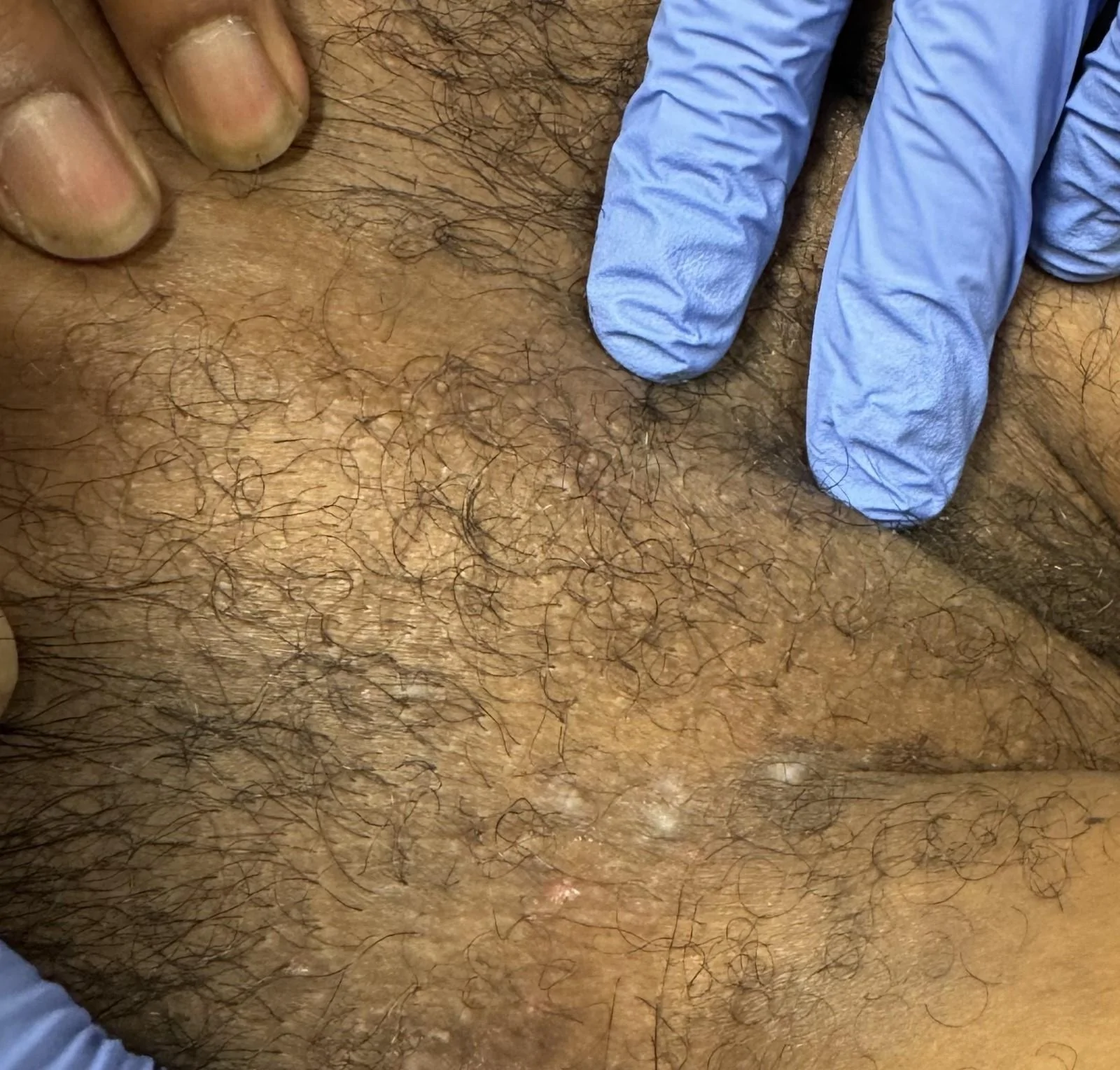
Clinical photograph of the patient’s groin showing multiple annular plaques with raised edges. This finding, along with equivocal fluorescence under a Wood’s lamp, initially suggested a possible fungal (tinea) coinfection.

The genitourinary examination revealed multiple shallow, tender, vesiculopustular lesions on the glans penis and shaft. Bilateral, tender inguinal lymphadenopathy was palpable. At the base of the penis and extending into the crural folds, annular plaques with raised, scaling edges were noted (Figure 1). Examination under a Wood’s lamp revealed equivocal fluorescence, initially raising suspicion for a fungal superinfection.

The rectal examination was notable for two superficial longitudinal ulcerations at the anal verge (Figure 3). These lesions clinically resembled anal fissures but were associated with significant tenderness and mild surrounding erythema. No hemorrhoids or active bleeding were visualized.

**Figure 2.**
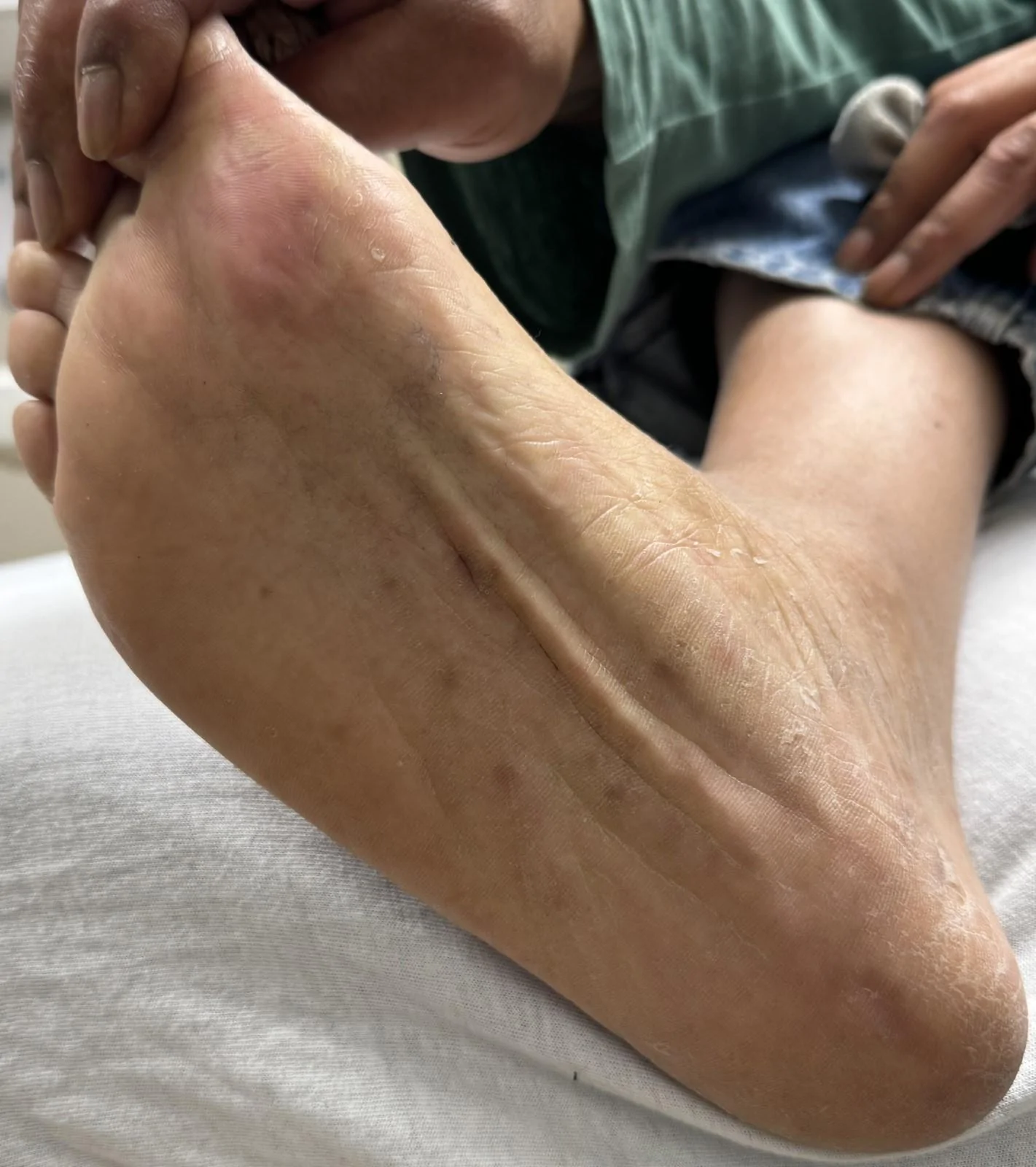
Plantar surface of the patient’s foot demonstrating numerous round to oval erythematous macules and papules. Some lesions presented with central dusky zones and peripheral erythema, consistent with the targetoid lesions characteristic of erythema multiforme.

Dermatologic examination of the extremities revealed numerous erythematous macules and papules on the palms and soles. Several lesions on the plantar surfaces exhibited a targetoid appearance, characterized by a central dusky zone surrounded by a pale edematous ring and an erythematous border (Figure 2).

**Figure 3.**
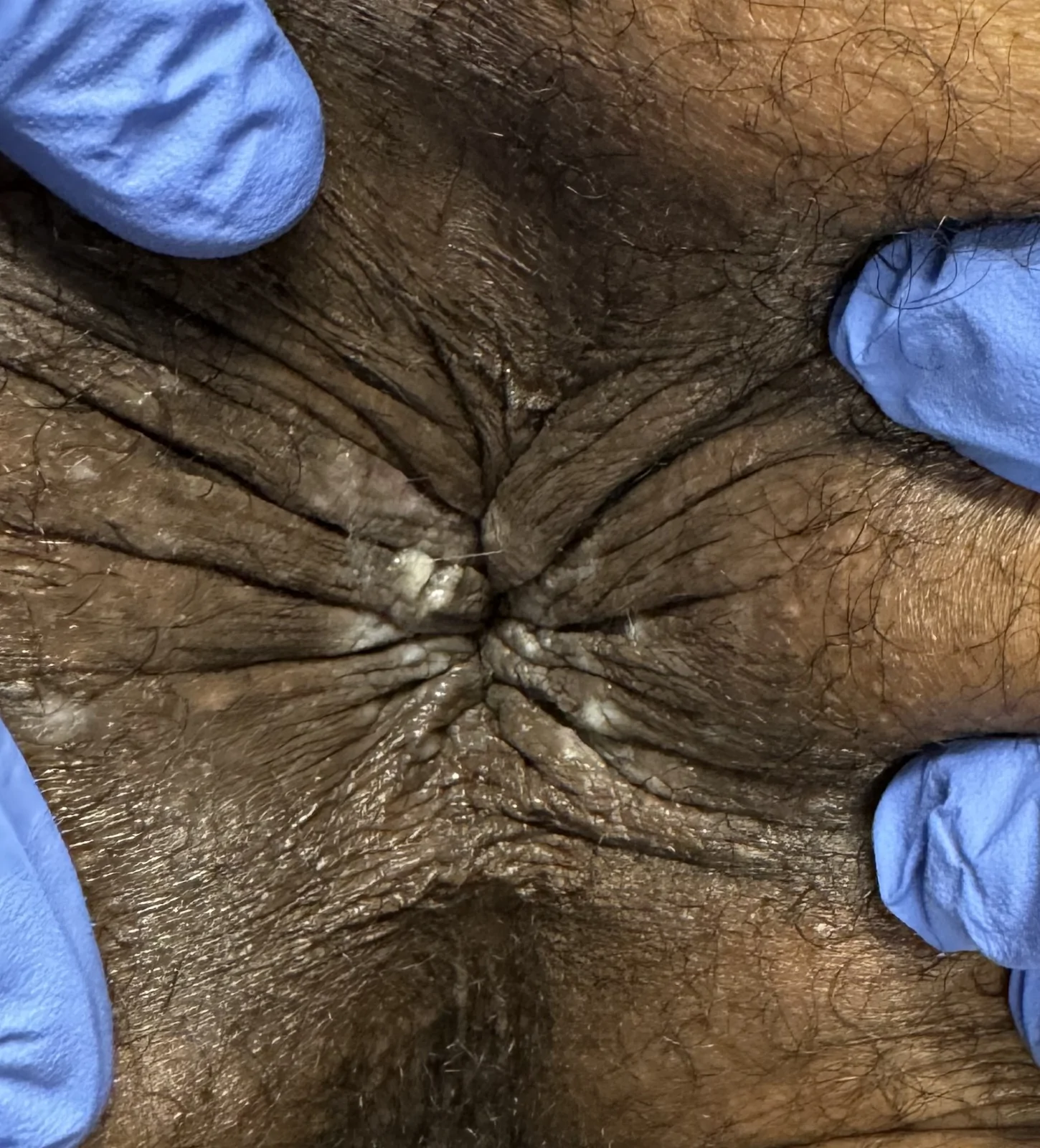
Perianal examination revealing two superficial ulcers at the anal verge with surrounding erythema. These lesions were clinically mistaken for anal fissures but were ultimately identified as manifestations of the HSV-2 infection.

**Diagnostic assessment:** Given the combination of genital ulcers and a palmoplantar rash in a high-risk patient, the pre-test probability for secondary syphilis was high. The differential diagnosis included disseminated gonococcal infection, reactive arthritis, chancroid, and HSV with EM (Table 1).

**Table 1.** Differential Diagnosis of Genital Ulcers with an Acral Rash

**Differential Diagnosis**	**Key Features**	**Distinguishing Points**
**Secondary syphilis**	Generalized rash often involving palms and soles; mucous patches; condyloma lata; systemic symptoms (fever, lymphadenopathy). Genital lesions (primary chancre or condyloma lata) may be present but often painless.	Palmar/plantar rash can appear coppery or scaly, not typically “target” lesions. Genital ulcer of primary syphilis is classically solitary and painless (though variations occur). Serologic tests (RPR, treponemal antibody) are usually positive in the secondary stage.
**Genital HSV infection**	Painful clusters of vesicles or ulcers on genital or anal area; may recur. Often associated with localized pain, burning, with or without fever. Typical lesions heal with crusting.	Lesions are usually painful and in crops. HSV can be confirmed by PCR or culture from lesions. Unlike syphilis, HSV does not typically cause a diffuse rash (any widespread eruption suggests coexistent EM or dissemination).
**HSV with erythema multiforme**	Target lesions on hands, feet, or elsewhere following an HSV outbreak. Target lesions have concentric rings (dark center, pale intermediate ring, outer red ring). May have some lesions with central blister or crust.	EM lesions are immune-mediated and not infectious. Diagnosis is clinical. HSV is a known trigger. Syphilitic rashes can mimic but usually lack classic target appearance.
**Reactive arthritis (Reiter’s syndrome)**	Triad: arthritis, urethritis, conjunctivitis. Cutaneous: keratoderma blennorrhagica (hyperkeratotic pustules on palms/soles), circinate balanitis. Often follows *Chlamydia* infection.	Genital lesions usually painless. Associated with joint pain and eye symptoms. Palmar/plantar lesions are wart-like. Chlamydia testing may help. HSV PCR is negative.
**Disseminated gonococcal infection (DGI)**	Dermatitis, migratory polyarthralgia, tenosynovitis. Few pustular or vesicular lesions on extremities (including palms). Genital symptoms minimal or absent.	Rash is sparse and systemic signs may be present. Confirmed via culture or NAAT from mucosal or blood specimens. Prominent genital ulcers are atypical.
**Hand-foot-and-mouth disease**	Coxsackievirus infection, usually pediatric. Fever, oral erosions, and vesicular palm/sole lesions.	Genital lesions rare. Epidemiology, age, and exposure history help. PCR from throat or lesions confirms enterovirus.
**Chancroid** (*Haemophilus ducreyi*)	Painful genital ulcer(s) with ragged undermined edges, suppurative inguinal nodes. Rare in developed countries.	Deep purulent ulcers. Confirmed via culture or PCR. Rash on hands/feet is absent.
**Others (e.g., Behçet’s, SJS, pemphigus syphiliticus)**	Rare causes of genital ulcers and palm involvement. Behçet’s: oral/genital ulcers + uveitis. SJS: mucosal erosion + drug trigger. Pemphigus: bullae.	Ruled out by mucosal sparing, lack of drug history, and systemic features. Important to consider if refractory.

Baseline laboratory investigations were obtained:

- **Complete Blood Count (CBC):** White blood cell count 6.8 x 10^3/uL (within normal limits), no leukocytosis.
- **Comprehensive Metabolic Panel (CMP):** Within normal limits; creatinine 0.9 mg/dL.
- **HIV 1/2 Ag/Ab combo:** Non-reactive.
- **Rapid Plasma Reagin (RPR):** Non-reactive.
- **Gonorrhea/Chlamydia NAAT (urine):** Negative.
- **HSV 1 & 2 Polymerase Chain Reaction (PCR):** Swabs were obtained from the penile lesions.

**Management** Pending laboratory confirmation, a syndromic management approach was utilized to cover the most dangerous treatable etiology. The patient received empiric treatment for secondary syphilis with Benzathine penicillin G 2.4 million units intramuscularly. He was also provided doxycycline 100 mg orally twice daily for 14 days to cover potential lymphogranuloma venereum or atypical pathogens.

**Follow-up and outcomes** The patient returned for follow-up 7 days later reporting minimal improvement in the genital lesions and persistence of the rash. At this time, the HSV PCR results returned positive for HSV-2. The RPR remained non-reactive, effectively ruling out syphilis given the duration of symptoms.

The diagnosis was revised to recurrent genital HSV-2 with secondary erythema multiforme and HSV proctitis. The patient was started on high-dose valacyclovir (1 gram orally twice daily) for 10 days. At a subsequent follow-up visit 14 days later, the genital ulcers had re-epithelialized, the anal pain had resolved, and the palmoplantar rash had faded completely. He was counseled on suppressive antiviral therapy (valacyclovir 500 mg daily) to reduce the frequency of recurrences and the risk of future EM episodes.

## DISCUSSION

This case illustrates the diagnostic pitfalls of “pattern recognition” in sexually transmitted infections. The classic teaching—that a rash on the palms and soles implies syphilis until proven otherwise—overshadowed the morphological nuances of the patient’s presentation. While secondary syphilis can present with papulosquamous lesions on the extremities (clavi syphilitici), true targetoid lesions are pathognomonic for erythema multiforme.^6,8^

HSV is the single most common infectious etiology of erythema multiforme, accounting for up to 70-80% of cases (often called HAEM - Herpes Associated Erythema Multiforme).^2,9,10^ The pathophysiology involves a type IV hypersensitivity reaction where CD4+ Th1 cells transport HSV DNA fragments to distant skin sites (like the palms), triggering an inflammatory cascade. This results in the confusing clinical picture of a “syphilis-like” distribution caused by a herpes virus.^9,10^

Furthermore, the patient’s anal symptoms were initially dismissed as fissures. Anal fissures are typically caused by trauma or hard stools and are located at the posterior or anterior midline. However, HSV is a well-documented cause of proctitis and perianal ulcerations, particularly in men who have sex with men (MSM) or via autoinoculation.^3,4,11^ Winceslaus et al. previously described genital herpes masquerading as anal fissures, noting that herpetic “fissures” are often multiple, lateral, and associated with inguinal adenopathy, consistent with our patient’s exam.^3^

### Osteopathic Implications

From an osteopathic perspective, this case highlights the importance of treating the whole patient—body, mind, and spirit—rather than focusing solely on the cutaneous manifestations. The patient’s recurrent, undiagnosed pain and the social stigma of a “syphilis” diagnosis likely contributed to significant psychological distress and somatic dysfunction. The lymphatic congestion noted in the inguinal region (adenopathy) represents a disruption in the body’s homeostatic mechanisms. An osteopathic approach would not only target the viral etiology with pharmacotherapy but also address the somatic components. Lymphatic pump techniques or pedal pump techniques could be considered (once acute local infection is controlled) to augment lymphatic return and immune response.^12^ Furthermore, addressing the patient’s visceral somatic reflexes—where rectal pain may contribute to pelvic floor hypertonicity and constipation—is vital for holistic recovery. By utilizing definitive testing (PCR) to provide an accurate diagnosis, the physician alleviates the patient’s anxiety regarding syphilis and allows for a targeted treatment plan that restores the body’s structure-function relationship.

### Limitations

This report represents a single case study. While the clinical diagnosis of EM was strong based on morphology and temporal association with HSV, a skin biopsy was not performed to histologically confirm EM. Additionally, long-term follow-up beyond one month was not available to assess the efficacy of suppressive therapy in preventing EM recurrence.

### Conclusion

Atypical presentations of HSV-2 can mimic a wide range of dermatologic and systemic conditions. The combination of genital ulcers and an acral rash should prompt consideration of HSV-associated erythema multiforme, not just syphilis. Clinicians should maintain a low threshold for utilizing HSV PCR testing in patients with “anal fissures” or polymorphic rashes that do not respond to standard empiric antibiotic therapy. Early identification prevents unnecessary antibiotic use and alleviates patient morbidity through targeted antiviral treatment.

### Patient Consent

Written informed consent was obtained from the patient for publication of this case report and accompanying images. A copy of the written consent is available for review by the Editor-in-Chief of this journal.
